# Molecular remission after combination therapy with blinatumomab and ponatinib with relapsed/refractory Philadelphia chromosome-positive acute lymphocytic leukemia: two case reports

**DOI:** 10.1186/s13256-021-02771-z

**Published:** 2021-03-25

**Authors:** Junichiro Yuda, Nobuhiko Yamauchi, Ayumi Kuzume, Yong-Mei Guo, Nobue Sato, Yosuke Minami

**Affiliations:** 1 Department of Hematology and Oncology, National Cancer Center Hospital East, 6-5-1, Kashiwano-ha, Kashiwa, 277-8577 Japan; 2 Pharmaceutical Department, National Cancer Center Hospital East, Kashiwa, Japan

**Keywords:** Relapsed/refractory Philadelphia chromosome-positive acute lymphoblastic leukemia, Blinatumomab, Ponatinib, Tyrosine kinase inhibitor, *BCR–ABL* compound mutations

## Abstract

**Background:**

The outcomes of Philadelphia chromosome-positive (Ph+) acute lymphoblastic leukemia (ALL) can improve with allogeneic hematopoietic stem cell transplantation (HSCT) during the first complete remission after treatment with a tyrosine kinase inhibitor (TKI) combined with chemotherapy. However, frail patients who are not eligible for allogeneic HSCT or those with TKI-resistant mutations within the BCR–ABL kinase domain have a poor clinical course. Blinatumomab (BLIN) is a bispecific T-cell engager antibody construct that directs cytotoxic T cells to CD19-expressing B-ALL cells. To date, only a few studies have shown the safety and efficacy of Blinatumomab (BLIN) + TKI combination therapy for relapsed/refractory (R/R) Ph+ ALL. Here we report the case of two patients with R/R Ph+ ALL who were treated with BLIN + TKI with durable molecular response.

**Case presentation:**

Patient 1: A 69-year-old Japanese male with R/R Ph+ ALL was treated with conventional chemotherapy and dasatinib in April 2016. In May 2018, he developed molecular relapse due to the acquisition of T315I during dasatinib maintenance therapy. Thereafter, he achieved molecular complete remission (mCR) after switching from dasatinib to ponatinib. However, he developed a second relapse after the emergence of triple compound mutations (G250E/D276G/T315I) in November 2018. He subsequently received a total of nine cycles of BLIN and ponatinib combination therapy, which resulted in sustained mCR without any adverse events. Patient 2: A 69-year-old Japanese female with R/R Ph+ ALL was treated with chemotherapy and imatinib in April 2008. She developed molecular relapse due to the emergence of the T315I mutation in October 2017. She achieved mCR after switching from imatinib to ponatinib. However, she developed a second relapse after acquiring *ABL* exon4 skipping in addition to T315I. She subsequently received a total of seven cycles of BLIN and ponatinib combination therapy, which resulted in sustained mCR.

**Conclusion:**

In our two cases, BLIN + ponatinib combination therapy was highly effective for R/R Ph+ ALL without any incidence of severe adverse events. Further studies with larger cohorts are warranted to validate the safety and efficacy of this potent combination therapy.

## Background

Philadelphia chromosome-positive (Ph+) acute lymphoblastic leukemia (ALL) accounts for approximately 25% of all adult B-precursor ALL cases. This condition is characterized by a reciprocal t(9;22) translocation that generates BCR–ABL, a chimeric fusion protein, with constitutively upregulated tyrosine kinase activity. The presence of the Ph chromosome has been associated with an extremely poor prognosis [1]. However, the response to treatment and survival outcomes have improved significantly over the last decade [2–4]. The combination of BCR–ABL tyrosine kinase inhibitors (TKIs) and chemotherapy has substantially improved the outcome of patients with Ph+ ALL [2–4]. Although combination chemotherapy with imatinib or dasatinib is effective, the 3-year event-free survival and overall survival rates for adult patients with Ph+ ALL are approximately 40% and 60%, respectively [2, 3]. In developing a novel therapeutic strategy for patients with Ph+ ALL, the acquisition of TKI resistance induced by point mutations within the BCR–ABL kinase domain (KD) should be addressed [5]. The T315I mutation, which is highly resistant to imatinib and second-generation TKIs, is a major cause of recurrence in patients with Ph+ ALL [5]. Ponatinib, a third-generation TKI, is a potent BCR–ABL inhibitor in leukemia patients with both wild-type and BCR–ABL mutations, including T315I [6]. However, a T315I-positive clone acquires an additional resistance mutation, resulting in high resistance even to ponatinib. Therefore, a new treatment strategy for eliminating such clones with double or triple compound mutations should be developed [7]. Blinatumomab (BLIN) is a bispecific T-cell engager antibody construct that directs cytotoxic T cells to CD19-expressing B cells [8–10]. BLIN has single-agent activity in patients with B-ALL with minimal residual disease (MRD) during the first complete remission (CR) or thereafter and in those with relapsed/refractory (R/R) B-ALL, including R/R Ph+ ALL [8, 9]. BLIN monotherapy has therapeutic effects on immunological outcomes regardless of the BCR–ABL status, even with the presence of the T315I mutation. Moreover, patients with Ph+ ALL who receive this treatment achieve hematological or molecular remission [8]. This result indicates that BLIN can eliminate highly TKI-resistant leukemic clones with double or triple compound mutations. A few studies have shown that TKI + BLIN combination therapy is safe and effective [11, 12]. This combination therapy is considered an extremely reasonable therapeutic strategy due to its mechanism of action. Herein, we report two patients with TKI-resistant R/R Ph+ ALL who achieved molecular remission after receiving BLIN + ponatinib combination therapy.

## Case presentation

### Patient 1

**Fig 1:**
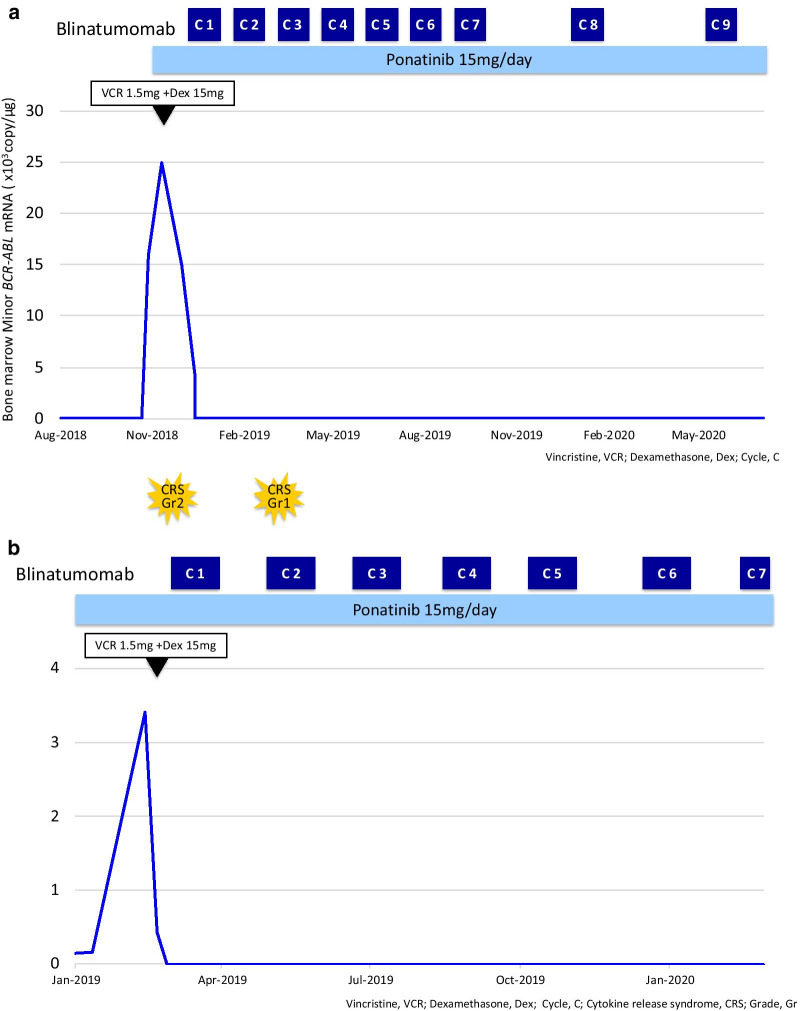
The clinical course of two cases under BLIN + ponatinib combination therapy. The clinical courses of cases 1 and 2 are shown in **a**, **b**, respectively. In each chart, the vertical axis and horizontal axis show the amount of *BCR–ABL* (%) and the time point, respectively. *VCR* vincristine, *dex* dexamethasone, *C* cycle, *CRS* cytokine release syndrome, *Gr* grade

A 69-year-old Japanese male was diagnosed with Ph+ ALL in April 2016 and was enrolled in a phase 2 clinical trial (JALSG Ph ALL213 study, UMIN000012173). He received combination chemotherapy as induction therapy and dasatinib 100 mg/day in April 2016. Then, molecular CR (mCR) was achieved after induction therapy. Consolidation therapy was initiated in June 2016 and was continued until October 2016. He persistently achieved mCR. From November 2016 to January 2018, 12 courses of maintenance therapy with vincristine, prednisolone, and dasatinib were administered. Then, dasatinib monotherapy 140 mg was continued until February 2018. Unfortunately, the copy number of minor *BCR–ABL* increased to 140,000 copies in May 2018. Dasatinib was switched to ponatinib 15 mg/day due to the emergence of the T315I mutation. While mCR was achieved with ponatinib, the copy number of minor *BCR–ABL* again increased to 25,000 copies after the emergence of triple compound mutations (G250E/D276G/T315I) in November 2018 (Fig. 1a, Table 1). In December 2018, vincristine 1.5 mg and dexamethasone 15 mg were administered and the copy number of minor *BCR–ABL* decreased to 2000. BLIN was initiated at a dose of 9 μg/day. Then, the dose was gradually increased to 28 μg/day. Ponatinib 15 mg was continued during BLIN treatment. No adverse events, such as cytokine release syndrome (CRS), immune effector cell-associated neurotoxicity syndrome (ICANS), or cardiovascular events, were observed during BLIN + ponatinib combination therapy. MRD was evaluated by real-time quantitative polymerase chain reaction (RQ-PCR) of *BCR–ABL* transcript and multicolor flow cytometry (leukemic population: CD10^+^19^+^20^dim^34^+^45^dim^HLA-DR^+^). After one cycle of combination therapy, neither RQ-PCR nor multi-colored flow cytometry could not detect MRD in these patients. In total, nine cycles of BLIN + ponatinib combination therapy were carried out until January 2020. Since February 2020, mCR has been sustained after switching the combination therapy to ponatinib monotherapy (Fig. 1a). This result indicated that the leukemic clone with triple compound mutation was completely eradicated with BLIN + ponatinib combination therapy.

**Table 1:** Laboratory data

Peripheral blood	Case 1	Case2		Chemistry	Case 1	Case 2	
White Blood Cell	6,100	14,300	/μl	Total Protein	7.2	7.6	g/dl
Neutrophil	79.3	80.7	%	Albumin	4.9	3.9	g/dl
Lymphpocyte	14.7	15.2	%	Uric Acid	6.6	5.4	mg/dl
Monocyte	5.6	3.1	%	Creatinine	1.02	0.75	mg/dl
Eosinophil	0.2	0.4	%	Total Bilirubin	0.2	0.2	mg/dl
Basophil	0.2	0.6	%	AST	19	21	IU/l
Red Blood Cell	409	472	×10^4^/μl	ALT	14	15	IU/l
Hemoglobin	12.6	13.5	g/dl	ALP	299	361	IU/l
Hematocrit	38.4	41.3	%	Bone marrow			
MCV	93.9	87.5	fl	Cellularity	Normal	Normal	
MCH	30.8	28.6	pg	Leukemia cells	8.2	0	%
MCHC	32.8	32.7	%	G-Banding	46,XY[20]	46,XX[20]	
Platelet	29.2	13.5	×10^4^/μl	Minor *BCR–ABL* copy number	25,000	330	copies

*MCV* Mean Corpuscular Volume, *MCH* Mean Corpuscular Hemoglobin, *MCHC* Mean Corpuscular Hemoglobin Concentration, *AST* Aspartate Aminotransferase, *ALT* Alanine Aminotransferase, *ALP* Alkaline Phosphatase

### Patient 2

A 69-year-old Japanese female was diagnosed with Ph+ ALL in April 2008, and she was subsequently registered in the JALSG Ph ALL202 trial until June 2010. The patient received induction therapy, consolidation therapy, and then maintenance therapy [13]. She consistently achieved mCR while on treatment with imatinib 600 mg/day after maintenance therapy. However, in October 2017, she developed molecular relapse with a minor *BCR–ABL* copy number of 440. ABL sequence analysis revealed that the leukemic clone possessed the T315I mutation. Therefore, imatinib was switched to ponatinib 15 mg/day. Thereafter, the patient achieved mCR again while on ponatinib treatment. However, in February 2019, she again developed molecular relapse with a minor *BCR–ABL* copy number of 330 (Fig. 1b, Table 1). The direct sequence of *ABL* revealed that the leukemic clone acquired exon4 skipping in addition to T315I mutation. Thereafter, in addition to oral ponatinib 15 mg, continuous infusion of BLIN 9 μg/day was started. However, the patient experienced grade 2 CRS on day 3 of cycle 1. To manage grade 2 CRS, the patient required dexamethasone 19.8 mg. Then, BLIN was promptly restarted at the same dose, and mCR was achieved after the end of cycle 1. After one cycle of combination therapy, neither RQ-PCR nor multicolor flow cytometry could detect MRD. In cycle 2, the patient presented with grade 1 CRS while the dose of BLIN was increased up to 28 μg/day. Then, the dose was reduced from 28 to 9 μg/day, and 9 μg/day of BLIN + ponatinib 15 mg/day was continued. The patient safely completed seven cycles of BLIN treatment without any adverse events such as CRS or ICANS during the whole clinical course. In addition, she achieved mCR while on ponatinib monotherapy after seven cycles of BLIN + ponatinib combination therapy (Fig. 1b).

## Discussion and conclusion

This case report showed that BLIN + ponatinib combination therapy was effective in two patients with RR Ph+ ALL who were not eligible for transplantation. In the pivotal phase 2 ponatinib Ph+ ALL and CML evaluation (PACE) trial of single-agent ponatinib in patients with relapsed/refractory Ph+ leukemia, the complete cytogenetic response and MR4.5 rates were 72% and 37%, respectively [6]. In the BLIN in patients with minimal residual disease of B-precursor acute lymphoblastic leukemia (BLAST) study of patients with Philadelphia-negative ALL who presented with MRD treated with BLIN, the MRD negativity rate after one cycle was 78% [9, 10]. ALCANTARA is a single-arm phase 2 study of 45 patients with relapsed/refractory Ph+ ALL who previously experienced treatment failure with TKIs. In the ALCANTARA trial, the combined CR/hematological CR (CRh) rate of BLIN monotherapy after the first two cycles was 36% [8]. Approximately 88% of patients with CR/CRh achieved MRD negativity even if they possessed KD mutations within the BCR–ABL KD [8].

Various groups have demonstrated the efficacy and safety of BLIN + TKI combination therapy. Assi *et al*. showed that 9 (75%) of 12 patients with RR Ph+ ALL/RR chronic myelogenous leukemia-lymphoid blast crisis who received BLIN + TKI (*n* = 8, ponatinib; *n* = 3, dasatinib; and *n* = 1 bosutinib) achieved molecular response [11]. Moreover, eight of nine patients with RR Ph+ ALL who received BLIN + TKI (*n* = 5, ponatinib; *n* = 4, dasatinib; *n* = 1, nilotinib; and *n* = 1, imatinib) achieved molecular response [12]. Recently, dasatinib + blinatumomab and blinatumomab + ponatinib combination therapy showed high efficacy in patients with newly diagnosed and RR Ph+ ALL, respectively [14, 15]. The emergence of various clones based on the multi-blanching model contributes to the recurrence mechanism of ALL [16]; therefore, the combined use of multiple drugs with different mechanisms of action leads to overcoming the resistance of RR Ph+ ALL. Based on these results, blinatumomab + ponatinib combination therapy was selected in our two cases. In the current report, triple compound mutations (G250E/D276G/T315I) (case 1) and T315I/ABL exon4 skipping (case 2) [17] were observed. However, both patients achieved and experienced sustained mCR after receiving BLIN + TKI combination therapy. ALL clones comprise various clones based on the multi-branching model. Hence, ponatinib can play a role in preventing recurrence via the suppression of ponatinib-susceptible clones after the eradication of TKI-resistant clones with BLIN [16]. Both patients were over 65 years of age, and they did not agree to receive allogeneic hematopoietic stem cell transplantation (HSCT) due to the high rate of transplant-related mortality. Therefore, blinatumomab + ponatinib combination therapy was selected, and both patients have maintained long-term molecular remission. In patients with RR Ph+ ALL who are eligible for allogeneic HSCT, the combination of blinatumomab + tyrosine kinase inhibitor combination therapy with allogeneic transplantation or anti-CD19 chimeric antigen receptor (CAR-T) may further improve treatment outcomes.

BLIN causes adverse events such as CRS and ICANS via CD3-positive lymphocyte activation and cytokine overproduction. A few studies have shown that the incidence of CRS after BLIN + TKI combination therapy and BLIN monotherapy was similar [11, 12]. In the current study, one patient developed grade 2 CRS. However, this patient (case 2) recovered with the administration of dexamethasone. The dose of ponatinib is significantly associated with the occurrence of arterial occlusive events (AOEs) [6]. Assi *et al*. reported that some patients who had received BLIN + TKI combination therapy developed transaminitis [11]. In order to reduce the risk of developing adverse events associated with vascular occlusion, we measured the blood concentration of ponatinib using high-performance liquid chromatography during treatment [18]. Based on these data, both patients received ponatinib treatment at optimal concentrations (> 23 ng/ml) [18]. Since these patients also used with fluconazole, an antifungal drug that has a CYP3A4 inhibitory effect, they were treated with optimal blood concentration levels even at a low dose of ponatinib 15 mg/day. In the current study, the two patients tolerated the combination of BLIN + ponatinib 15 mg daily well, and there was no incidence of transaminitis or AOEs [6].

These clinical data support the notion that BLIN + ponatinib combination therapy is safe and can be well tolerated. The current studies on the safety and efficacy of BLIN + ponatinib combination therapy only included small cohorts. Hence, further studies with larger cohorts should be conducted to validate the safety and efficacy of this potent combination therapy.

## Data Availability

The data used in this report are available from the corresponding author on reasonable request.

## Abbreviations

ALL: Acute lymphoblastic leukemia
AOEs: Arterial occlusive events
BLIN: Blinatumomab
CRS: Cytokine release syndrome
HSCT: Hematopoietic stem cell transplantation
ICANS: Immune effector cell-associated neurotoxicity syndrome
KD: Kinase domain
mCR: Molecular complete remission
MRD: Minimal residual disease
Ph+: Philadelphia chromosome-positive
R/R: Relapsed/refractory
TKI: Tyrosine kinase inhibitor
